# False-Positive Phencyclidine (PCP) Result on 11-Panel Urine Drug Screen (UDS) in a 17-Year-Old Adolescent with Long-Term Venlafaxine Use

**DOI:** 10.1155/2023/6666197

**Published:** 2023-06-26

**Authors:** Hyun Sue Kim, Aakash Jain, Anita S. Kablinger

**Affiliations:** ^1^Virginia Tech Carilion School of Medicine, Roanoke, VA, USA; ^2^Department of Psychiatry and Behavioral Medicine, Carilion Clinic, Roanoke, VA, USA

## Abstract

Venlafaxine is an antidepressant belonging to the class of serotonin–norepinephrine reuptake inhibitors that are US Food and Drug Administration-approved to treat and manage symptoms of depression, anxiety, and other mood disorders in adults. We describe an adolescent patient who likely had a false-positive phencyclidine result detected with an 11-panel urine drug screen in an outpatient setting of long-term use of therapeutic venlafaxine extended release for the treatment of recurrent major depressive disorder and generalized anxiety disorder. We believe that this may be the first published case report to characterize this phenomenon in a young patient in the absence of an acute overdose.

## 1. Introduction

Venlafaxine is an antidepressant that belongs to the class of serotonin–norepinephrine reuptake inhibitors that work by increasing levels of serotonin, norepinephrine, and dopamine by blockage of reuptake at the presynaptic terminal [1]. Venlafaxine is US Food and Drug Administration (FDA)-approved to treat and manage symptoms of major depressive disorder (MDD), generalized anxiety disorder (GAD), social anxiety disorder, and panic disorder in adults [2]. In 2020, venlafaxine was the 43rd most commonly prescribed drug in the United States [3]. Venlafaxine extended-release (XR) tablets are not FDA-approved for use in pediatric patients; thus, any clinical need must be weighed against the potential risks.

Phencyclidine (PCP) is a dissociative anesthetic that became popular for recreational use in the 1970s [4]. PCP use has rapidly increased in recent years; between 2005 and 2011, emergency department (ED) visits related to PCP have increased five to sixfold [5]. PCP blocks the reuptake of dopamine and norepinephrine, leading to various central nervous system manifestations such as hypertension, tachycardia, nystagmus, and violent behavior [6].

A review of the literature indicates rare cases where venlafaxine led to false-positive results when testing for PCP [1, 7–9]. The vast majority of cases occurred in adults; only one case described a pediatric case in a 13-year-old girl in the setting of overdose with a false-positive PCP urine immunoassay and a negative gas chromatography–mass spectrometry (GC–MS) for PCP [8]. Moreover, a myriad of other medications have been reported in the literature to potentially cause false-positive PCP results, such as dextromethorphan, ibuprofen, imipramine, diphenhydramine, ketamine, lamotrigine, and tramadol [10].

We describe an adolescent patient who likely had a false-positive PCP result detected with an 11-panel urine drug screen (UDS) in an outpatient setting of long-term use of therapeutic venlafaxine XR for the treatment of recurrent MDD and GAD. We believe that this may be the first published case report to characterize this phenomenon in a young patient in the absence of an acute overdose.

## 2. Case Presentation

JC, a 17-year-old female, began outpatient psychiatric care at the age of 14 for the management of recurrent MDD and GAD; she also has a past medical history of gastroesophageal reflux disease, dyspepsia, and dysmenorrhea. The interview was first conducted with the legal guardian of the patient, then with the patient alone.

JC previously failed a trial of sertraline due to perceptual disturbance intolerability. At 15 years of age, she presented to the ED following an intentional overdose of approximately 20 pills of venlafaxine XR 37.5 mg in the setting of a psychological stressor. She was admitted to inpatient psychiatry with a voluntary status; UDS at the time was unremarkable. Once her condition stabilized, she was titrated over the next 2 years to venlafaxine XR 150 mg for residual MDD symptoms. She was also on hydroxyzine 10 mg as needed for anxiety attacks.

At age 17, a routine UDS returned positive for PCP over the cutoff concentration of 25 ng/mL; all other components were negative. The patient endorsed episodic marijuana use and experimentation with vaping in the past but denied any other substance misuse, including any PCP use. Other lab work, including complete blood count, comprehensive metabolic panel, thyroid-stimulating hormone, hemoglobin A1C, lipid profile, Vitamin D, and urinalysis, were grossly unremarkable.

## 3. Discussion

There is some evidence that venlafaxine's major metabolite, O-desmethylvenlafaxine (ODV), is in part associated with producing false-positive PCP results. One study prepared concentrations of venlafaxine or ODV in the laboratory, which resulted in positive results for PCP, indicating that there is some cross-reactivity between the drug and the metabolites with the PCP assay reagent [7, 9]. The FDA warns that there is a possibility of receiving a false positive PCP result even after discontinuing venlafaxine for several days [2, 9, 11]. Our case report is limited in that we did not conduct additional specific analytical testing, such as GC–MS, to confirm the absence of PCP.

The other case report published in the literature among pediatric patients occurred in the setting of acute overdose [8]. Our case presentation is unique in that it occurred in the absence of an acute overdose, and the medication was taken long-term. Our patient had a history of overdose with venlafaxine XR 2 years prior, with an unremarkable UDS, and denied recent misuse of prescribed medications or other substances.

JC was taking XR 150 mg venlafaxine pills, which is less than the outpatient maximum dose of 225 mg/day. Moreover, venlafaxine XR has a half-life of 5–7 hr and its metabolite, ODV, has a half-life of 11–13 hr. Despite the relatively short half-life, the patient still had a false-positive result for PCP. It is possible that the patient's chronic use of venlafaxine, in addition to the history of overdose with the same medication, could have predisposed her to experience a false-positive PCP result. Further research is warranted to better understand this mechanism.

While there is a possibility that there may have been PCP use by the patient, the false-positive PCP result and subsequent discussion can lead to the erosion of trust between the patient and the provider. Repeatedly asking about substance misuse in the absence of misuse can lead to continued mistrust. Patients are often in vulnerable states during their psychiatry visits since they share personal details about their lives that are seldom shared with others. Thus, we believe there is a need to warn patients regarding the association between venlafaxine XR use and false-positive PCP results as a part of transparent communication during patient-centered care.

## 4. Conclusion

Venlafaxine remains one of the most prescribed medications in the United States for the treatment of depression, anxiety, and other mood disorders. There is a need to further study the association between venlafaxine metabolism and PCP assay reagents commonly used in UDS, particularly in pediatric patients, to improve patient-centered care.

## Data Availability

No underlying data were collected or produced in this study.

## References

[1] Singh D., Saadabadi A. (2023). Venlafaxine. *StatPearls [Internet]*.

[2] USFDA (October 2015). Venlafaxine (marketed as Effexor) information. https://www.fda.gov/drugs/postmarket-drug-safety-information-patients-and-providers/venlafaxine-marketed-effexor-information.

[3] ClinCalc (August 2022). Venlafaxine drug usage statistics, United States, 2013–2020. https://clincalc.com/DrugStats/Drugs/Venlafaxine.

[4] Journey J. D., Bentley T. P. (2023). Phencyclidine toxicity. *StatPearls [Internet]*.

[5] Substance Abuse and Mental Health Services Administration (November 2013). The DAWN report: emergency department visits involving phencyclidine (PCP). https://www.samhsa.gov/data/sites/default/files/DAWN143/DAWN143/sr143-emergency-phencyclidine-2013.pdf.

[6] Bey T., Patel A. (2007). Phencyclidine intoxication and adverse effects: a clinical and pharmacological review of an illicit drug. *The California Journal of Emergency Medicine*.

[7] Sena S. F., Kazimi S., Wu A. H. B. (2002). False-positive phencyclidine immunoassay results caused by venlafaxine and *O*-desmethylvenlafaxine. *Clinical Chemistry*.

[8] Bond G. R., Steele P. E., Uges D. R. A. (2003). Massive venlafaxine overdose resulted in a false positive Abbott AxSYM® urine immunoassay for phencyclidine. *Journal of Toxicology: Clinical Toxicology*.

[9] Landy G. L., Kripalani M. (2015). False positive phencyclidine result on urine drug testing: a little known cause. *BJPsych Bulletin*.

[10] Michael Farley T., Anderson E. N., Feller J. N. (2017). False-positive phencyclidine (PCP) on urine drug screen attributed to desvenlafaxine (Pristiq) use. *BMJ Case Reports*.

[11] USFDAUS (2017). Effexor (venlafaxine HCl) tablets and Effexor XR (venlafaxine HCl) extended-release capsule. https://www.accessdata.fda.gov/drugsatfda_docs/label/2017/022104s015lbl.pdf.

